# Surgeon-modified fenestrated endograft for urgent an aortic arch aneurysm: case report

**DOI:** 10.1186/s13019-023-02102-x

**Published:** 2023-01-07

**Authors:** Vito Gallicchio, Danilo Barbarisi, Flavia Condò, Rosaria Sciarrillo, Loris Flora

**Affiliations:** 1 Vascular Surgery, Hospital of National Importance San Giuseppe Moscati, Via Contrada Amoretta, 83100 Avellino, Italy; 2 Department of Science and Technologies, University of Sannio, Benevento, Italy

**Keywords:** Aortic arch, Arch aneurysm, Carotid-carotid-succlavium bypass, Surgeon-modified fenestrated endograft, Hybrid procedure

## Abstract

We report the case of an endovascular repair of an aortic arch aneurysm by a surgeon-modified fenestrated endograft with a single fenestration in a high-risk patient unfit for open surgery. A patient of 84 years, chronic ischemic cardiopathic, suffering from prostate adenocarcinoma in chemotherapy treatment, came to our hospital for post-traumatic fracture of the right femur. During the hospitalization, the patient exhibited dysphonia and respiratory disorders for several days, therefore, the patient performed Computed Tomography Angiography (CTA) that found the presence of voluminous aneurysm of the aortic arch with a maximum diameter of about 74 mm. The patient was treated with a hybrid-staged procedure; in the first instance, with a carotid-carotid-succlavium bypass to preserve the cerebral and upper limb vascularization and then, the procedure was completed by implanting the surgeon-modified fenestrated endograft with stent delivery to the patient with a fenestration on the anonymous trunk. This surgeon-modified fenestrated endograft was created by modifying a standard endograft by a single fenestration following the three-dimensional reconstructions of the CTA images. The procedure was successfully completed and postoperative course was uneventful. Computed Tomography Angiography demonstrated the exclusion of the aneurysm, patency of the implanted endograft modules, and absence of signs of endoleaks and / or cerebral or medullary ischemic complications.

**Supplementary Information:**

The online version contains supplementary material available at 10.1186/s13019-023-02102-x.

## Background

Aortic arch aneurysm repair remains a major surgical challenge. Various strategies have been developed in order to limit the morbidity and mortality associated with open surgical repair, the major concern being neurologic morbidity with a reported rate of perioperative stroke ranging from 5 to 12% [1–4].

To further minimize the perioperative risks and the potential negative impact of these complex procedures on long-term outcomes of patients with aortic arch aneurysms, a concept of total endovascular repair for aortic arch disease has recently emerged.

The mortality and morbidity rates were reduced with the endovascular therapies with *custom-made* stent grafts (CSGs) in patients unfit for surgery [5, 6].

We present the case of a patient with a large saccular aneurysm of the aortic arch successfully treated with a physician-modified fenestrated stent-graft by a carotid-carotid-succlavium bypass.

Surgeon-Modified fenestrated endograft with a single fenestration are useful for treating distal arch pathology where the proximal landing zone commences at the distal margin of the Left Common Carotid Artery origin and blood flow into the Left Subclavian Artery needs to be preserved; however, such endografts could also be used to preserve flow into the Left Common Carotid Artery or the innominate artery if the more distal arch branches are revascularized by other means, such as bypass surgery.

Thoracic endovascular repair of an aortic arch aneurysm with a through technique may be considered for high-risk patients in an acute setting. In fact, recently, a new method for passing a deviation during treatment of an aortic arch aneurysm with a physician-modified fenestrated stent-graft was presented [7]. In fact, a 78-year-old man showed a rapidly expanding aortic arch aneurysm, and the limited landing zone required the use of a fenestrated/branched arch endograft. Physician-modified fenestrated stent-grafts are considered suitable for patients in an acute setting. The device became malrotated in a clockwise direction during deployment; therefore, cannulation of the first inner branch using a through-and-through wire technique was needed. The final angiogram showed a good result.

Thoracic endovascular repair of an aortic arch aneurysm using a through-and-through technique can be considered for high-risk patients in an acute setting.

## Case report

**Fig. 1:**
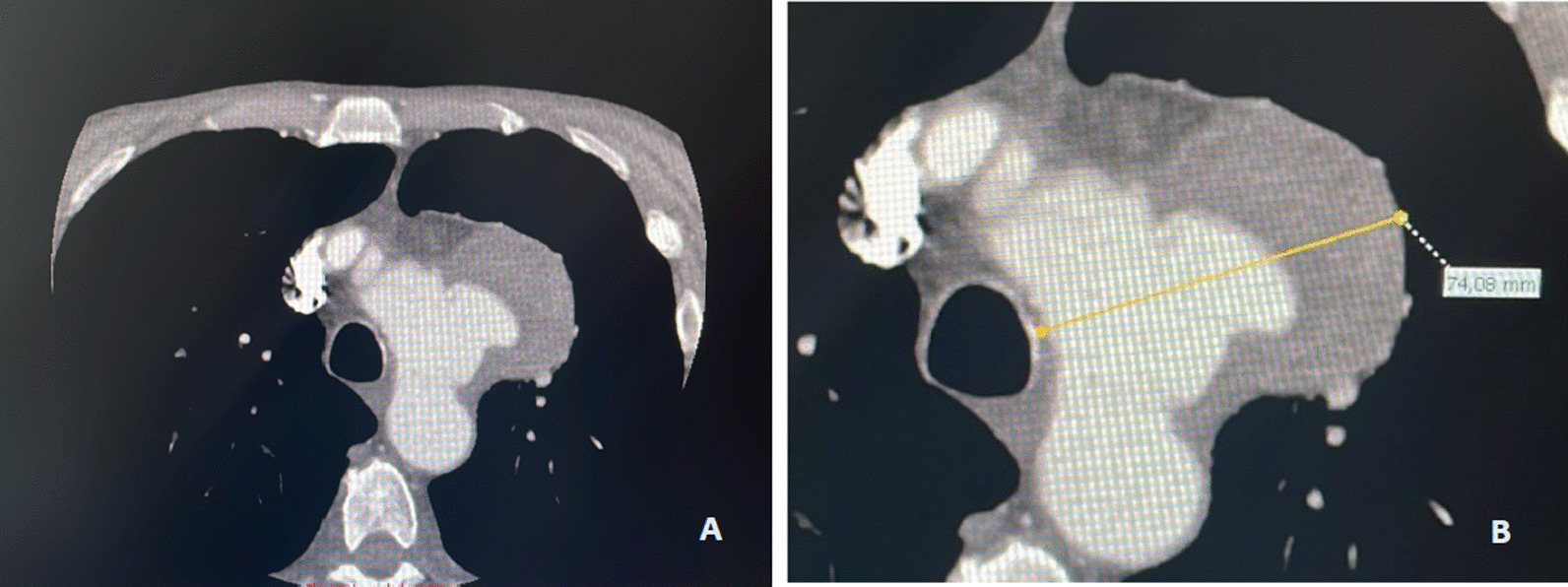
Pre-treatment figures from CTA showing aneurysm. A more detailed description in the text

An 84-year-old patient, chronic ischemic heart disease, suffering from prostate adenocarcinoma undergoing chemotherapy, came to the emergency room of our hospital for post-traumatic fracture of the right femur. The characteristics of the patient are showed in Additional file 1: Table S1 (Additional file). In addition, during the hospital stay, the patient presented with signs of dysphonia and dyspnea and respiratory disorders for several days, therefore, Computed Tomography Angiography (CTA) was performed. The CTA has been revealed the presence of a voluminous aneurysm of the aortic arch with a maximum diameter of about 74 mm (Fig. 1A–B). The saccular aneurysm involved the origin of the Left Subclavian Artery (LSA, diameter: 9 mm) and Left Common Carotid Artery (LCCA, diameter: 7 mm) and Right Common Carotid Artery (RCCA, diameter: 8 mm) in the absence of a sufficient proximal aortic neck downstream of the Brachio-Cephalic Trunk (BCT).

The anatomical details of the aorta-iliac and supra-aortic trunk (length of the ascending aorta: 89 mm between the sino-tubular junction and the anonymous trunk, and diameter of the ascending aorta: ascending aorta with a maximum diameter of 41 mm, anonymous trunk with a diameter ≤ 20 mm and sealing area ≥ 20 mm, Iliac tortuosity Index = 1) directed the O.U. of Vascular Surgery team toward a hybrid-stage procedure.

The patient was treated, in the first instance, with a carotid-carotid-succlavium bypass to preserve the cerebral and upper limb vasculature; subsequently, the procedure was completed with implantation of the surgeon-modified fenestrated endograft with delivery of the stent to the patient with a fenestration on the anonymous trunk. The surgeon-modified fenestrated endograft was created by modifying a standard endograft with a single fenestration following three-dimensional reconstructions of CTA images.

The major difficulty was the stability of the system. The stiffness of the vessels, due to the atherosclerotic disease, their tortuosity, and the acute angle between the anonymous and arch made it necessary to retrieve the guide with a telpher technique in order to stabilize the whole and thus achieve a precise release of the prosthetic modules.

**Fig. 2:**
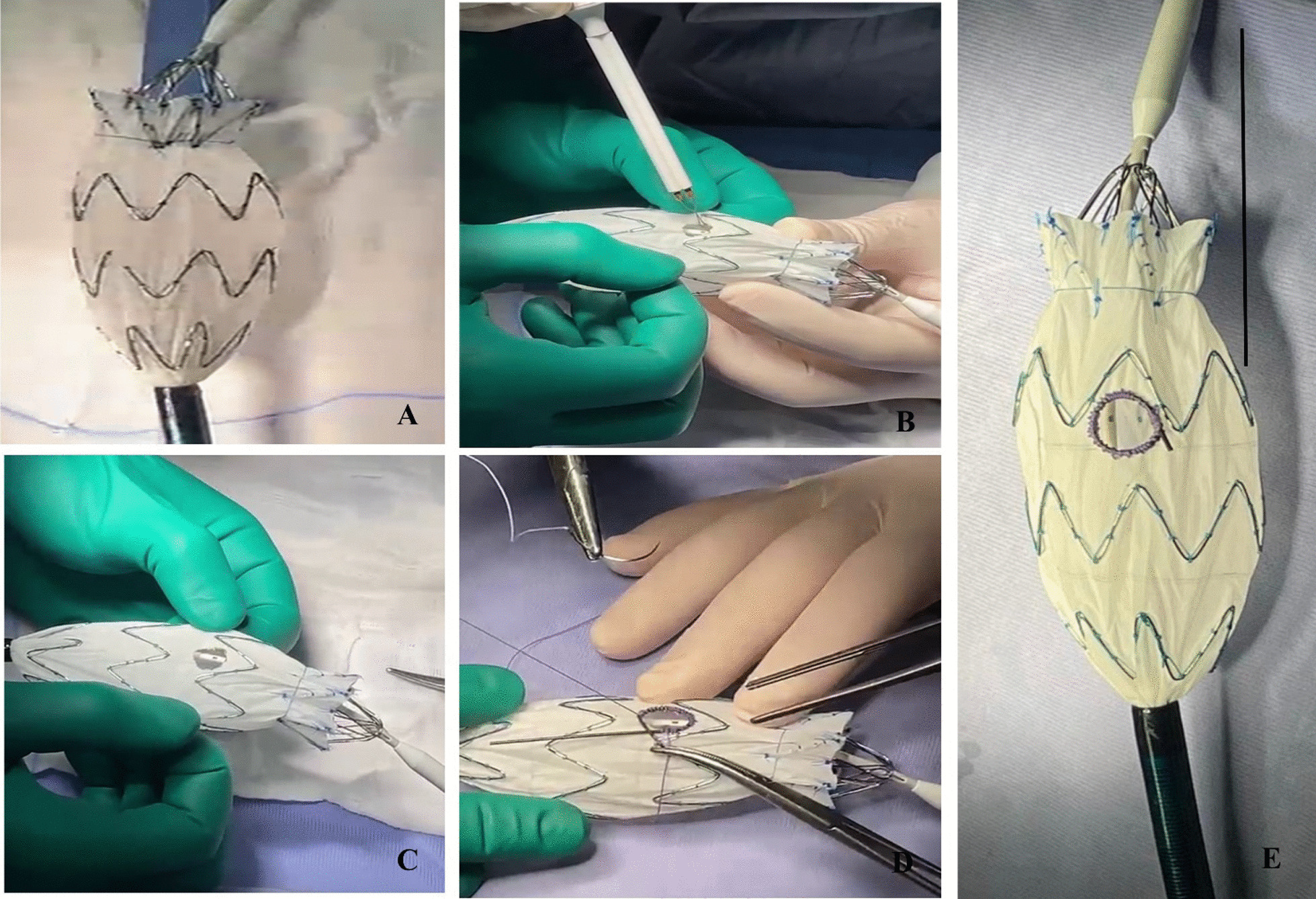
Photo of the Surgeon-Modified fenestrated endograft with a single fenestration for the Brachio-Cephalic Trunk (BCT) (**E**). **A** The standard iliac extension (Zenith Alpha™ Spiral-Z® Endovascular Leg -ZISL-20–77-Cook); **B**–**C** A single fenestration is using a thermal cautery instrument; **D** A radiopaque nitinol wire (3–0 Prolene wire) is sewn onto the edge of the fenestration. A more detailed description in the text

The aortic diameter did not create any particular difficulty while the anonymous diameter created problems because coated stents with comparable diameters with standard endografts were not available from O.U. Vascular Surgery Unit, and therefore, given the urgency of the case and it would not have been prudent to wait 30 days for the production of a custom-made endograft, the iliac extension (Zenith Alpha™ Spiral-Z® Endovascular Leg -ZISL-20–77-Cook) had to be modified (Fig. 2A).

A single fenestration (dm 1 cm) for the Brachio-Cephalic Trunk (BCT) of appropriate size and location is made in between the endograft stent struts, in line with the radiopaque mid-marker, using a thermal cautery instrument (Fig. 2B). Thereafter, a radiopaque nitinol wire (3–0 Prolene wire) is sewn onto the edge of the fenestration (Fig. 2C). In the arch endograft system described above, the fenestration is circular and is of comparable size to the target vessel; these features allow sealing in the ascending aorta (Fig. 2D).

The decision to implant a Cook's "series" aortic arch device was dictated first of all by the fact that the team of the O.U. of Vascular Surgery has decades of experience in using Cook's "series" device. In addition, in such a complicated and symptomatic case, the surgeons could not risk the success of the surgery and, given the urgency of the case, could not wait for the 30-day deadline for prosthesis construction, so they proceeded to modify Cook's "series" device.

Choosing a single fenestration instead of 2 and 3 would have made the surgery much longer and more complicated. Being familiar with traditional surgery, performing a carotid-carotid-succlavium bypass proved to be a quick operation with no major complications.

The procedure was carried out in the hybrid room by a team of vascular surgeons, assisted by interventional cardiologists following the three-dimensional reconstructions of CTA images. The surgical bypass was the versatile GORE® PROPATEN® Vascular Graft of 7 mm and it has been performed two days before of endovascular procedure. The origin of LCCA and LSA was excluded by surgical ligation. The type of projections is RAO15 and CRA10.

The cannulation of the fenestration was from below, with JR4 catheter and standard 0.035" hydrophilic guidewire through axillary artery. Given the tortuosity of the vessels, to have greater stability of the system, a parallel guide was placed, retrieved from the brachial access, using a telpheric technique. After that, from below, the iliac extension was placed at the level of the anonymous trunk. In this case, a tapered iliac extension was used, thus with a larger diameter end positioned in the anonymous and a smaller diameter end in the aortic arch. To adapt it to the anatomy, it was necessary to unsheathe the extension, shorten it, and sheath it in reverse to have a favorable release.

The total operation time was about 5 h. The procedure was performed in the operating room by a team of vascular surgeons, assisted by interventional cardiologists who inserted temporary cardiac pacing to produce bradycardia in the patient upon release of the endograft. To ensure access and navigability of the endograft, bilateral percutaneous access to the common femoral and surgical access from the right brachial artery were performed. Prosthesis opening, fenestration, iliac extension modification and recapture of the two-endograft modules were performed in about one and a half hours.

**Fig. 3:**
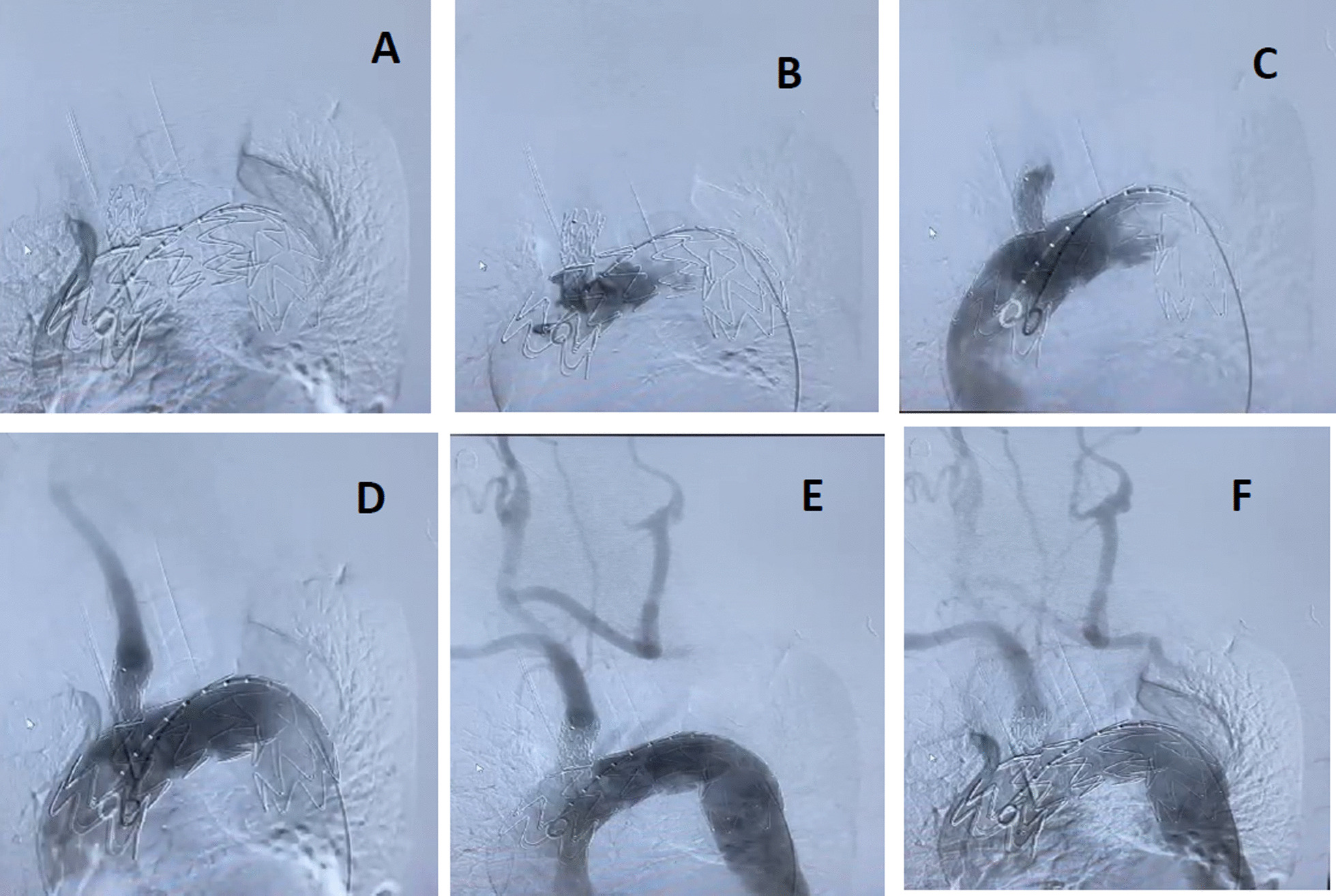
Intraoperative angiographic images in the positioning of the physician modified endograft in the aortic arch above the coronary plane and at the origin of the supra-aortic trunks **A**. Control angiography after complete release of the self-made endograft **B**. Selective angiographic control after delivery of the brachio-cephalic stem covered stent **C**, left common carotid artery **D** and by pass of the left subclavian artery **E**. The final angiography confirms the correct positioning of the physician modified endograft and the patency of the supra-aortic trunks **F**

Intraoperative and completion angiogram show exclusion of the aneurysm and preservation of all supraaortic branches with patency of the carotid-carotid-succlavium bypass and normal cerebral and upper limb vasculature (Fig. 3).

## Discussion

The recent guidelines for aneurysmal pathology of the aortic arch of the European Societies of Vascular Surgery and Cardio-Thoracic Surgery [12] recommend treatment endovascular through fenestrated endografts in specialized centers with high volume, for patients not suitable for traditional surgery, in the presence of specific anatomical features.

The development of endovascular technology has spurred another revolution in the management of aortic arch aneurysms [13].

However, CSGs have long manufacturing times and are therefore inappropriate for symptomatic and ruptured aortic arch aneurysm. In 2012, the first “*off-the-shelf*” multibranched endograft for endovascular aneurysm repair was approved in Europe. The initial clinical experiences showed interesting early results in both elective and urgent settings, but larger cohorts and longer follow-up are needed to verify this device. In fact, branched aortic endografts have been developed for this purpose and are currently undergoing clinical investigation [8–10]. The development of a device with a single internal branch allows complete endovascular treatment of the aneurysm of the aortic arch, reducing the time or the need of further anesthesia and the risks of damage to structures lymphatics, nerve perioperative hematomas. Precise deployment of these fenestrated arch endografts is important to correctly orient the fenestrations toward the branches for which they are intended. It is therefore desirable to have a system that allows full deployment of a fenestrated arch endograft immediately after introduction, facilitates correct orientation of the fenestrations, and minimizes subsequent manipulations to cannulate these openings [11].

Recent experiences of the literature have reported satisfactory technical results and clinical in the endovascular treatment of arch aneurysms aortic artery by branched endograft, with a mortality rate and perioperative cerebrovascular events ranging from 0.5 to 0.7%, respectively [8, 14–17]. The endograft most commonly implanted for this treatment is the one that uses two antegrade branches for the trunk anonymous and the Left Common Carotid Artery (CCS) with associated surgical revascularization of the Left Subclavian Artery (ASS) by carotid-subclavian bypass or transposition of the subclavian artery.

Recently, however, Gallitto et al. [9] underwent the endovascular treatment of an arch aneurysm aortic by means of custom made endograft with three internal branches for the supraortic trunks, in a patient at high risk for the conventional surgical treatment.

In this report case, instead, we describe the effective treatment of a voluminous aneurysm of the aortic arch via a hybrid-staged procedure, in a high-risk patient aged over eighty considered unsuitable for traditional surgery. This procedure was performed using a physician-modified endograft with a single fenestration that ensured the revascularization of the supra-aortic trunks. These data can be evaluated for a greater diffusion of the hybrid-staged procedure of aneurysms of the aortic arch and in the eventual creating an *off-the-shelf device* available for cases urgent or for bulky aneurysms where the risk of rupture during the customization times it is not negligible.

The use of physician-modified endograft in our case report is similar to case report present to Yang and Zhou [7]. They reported a novel means of overcoming a deviation when treating an aortic arch aneurysm with a physician-modified fenestrated stent-graft. Physician-modified fenestrated stent-grafts are considered suitable for patients in an acute setting.

## Conclusions

A physician-modified endograft with a single fenestration is a safe and effective option to guarantee a total endovascular repair of aortic arch aneurysm in high-risk patients in the presence of anatomical feasibility. Further evaluation is required to confirm these promising results. Therefore, thoracic endovascular repair of an aortic arch aneurysm using a hybrid-staged technique can be considered for high-risk patients in an acute setting.

## Supplementary Information

**Additional file 1: Table S1. **Characteristics of the patient.

## Data Availability

The dataset supporting the conclusions of this article is included within the article, and any other inquiry is available from the corresponding author on reasonable request.
