# Supplementary material for: Surgeon-modified fenestrated endograft for urgent an aortic arch aneurysm: case report
Source: J Cardiothorac Surg. 2023 Jan 7;18:7. doi: 10.1186/s13019-023-02102-x (PMC9824962; doi:10.1186/s13019-023-02102-x)
Supplement: Supplementary file 1 — Additional file 1: Table S1. Characteristics of the patient. [file 13019_2023_2102_MOESM1_ESM.docx]

**Table S1.** Characteristics of the Patient.

| *Patient Characteristics* | |
| --- | --- |
| Age, y | 84 |
| Hypertension^a^ | 1 |
| Diabetes | 0 |
| Dyslipidemia | 1 |
| Smoking | 0 |
| COPD | 1 |
| CAD | 1 |
| LV ejection fraction <40% | 0 |
| Prior aortic surgery | 0 |
| Renal insufficiency | 0 |
| eGFR, mL/min/1.73 m^2^ | 103 |
| ASA score | 2 |

Abbreviations: ASA, American Society of Anesthesiologists; CAD, coronary artery disease; COPD, chronic obstructive pulmonary disease; DAP, dose area product; eGFR, estimated glomerular filtration rate; LV, left ventricular; ^a^Systolic pressure >140 mm Hg or treatment.
